# Targeting LAIR1-mediated immunosuppression adds a new weapon to our immunotherapy arsenal

**DOI:** 10.1172/JCI194924

**Published:** 2025-08-15

**Authors:** Ezri P. Perrin, Hannah K. Dorando, Jacqueline E. Payton

**Affiliations:** Department of Pathology and Immunology, Washington University School of Medicine, St. Louis, Missouri, USA.

## Abstract

Leukocyte-associated Ig-like receptor 1 (LAIR1) is a collagen-binding inhibitory immune receptor that negatively regulates cellular activation. In this issue of the *JCI*, Tao et al. show that LAIR1-inhibitory signaling plays an important role in immunosuppressive M2-like tumor-associated macrophages (TAMs) in aggressive brain tumors. LAIR1 KO, antibody blockade, and an immunotherapy that incorporates a LAIR1-inhibitory module into a chimeric antigen receptor (CAR) all led to increased antitumor activity by CAR T cells, reduced M2-like TAMs, altered collagen networks, and increased survival rates in mouse tumor models. These findings demonstrate an innovative immunotherapeutic approach for cancer that leverages LAIR1 inhibition to combat multiple tumor immune evasion strategies.

## Tumor-associated macrophages and cancer immunotherapy resistance

Immunotherapies have emerged as highly promising new approaches to combat cancer; these include antibodies against programmed cell death protein 1 (PD-1), programmed death ligand 1 (PD-L1), and CTLA4 (checkpoint blockade), and modified immune cells that target tumors, exemplified by chimeric antigen receptor T (CAR T) cells (1, 2). Both therapy types depend on infiltration of activated effector immune cells into the tumor microenvironment (TME). However, many cancer types are resistant to currently available immunotherapies, or develop resistance after the initial response, likely due to a combination of mechanisms (3). In the present study, Tao et al. (4) sought strategies to simultaneously overcome multiple resistance mechanisms: the immunosuppressive effects of inhibitory immune receptors, tumor-associated macrophages (TAMs), and the collagen-rich tumor extracellular matrix, which promotes cancer cell growth while obstructing the entry of antitumor immune cells (5–7).

Leukocyte-associated Ig-like receptor 1 (LAIR1) is an inhibitory immune transmembrane receptor with an extracellular Ig-like domain, a transmembrane region, and an intracellular signaling region containing two cytoplasmic immunoreceptor tyrosine-based inhibitory motifs (ITIMs) (8). The Ig-like domains of LAIR1 and its secreted paralog, LAIR2, bind collagens and other proteins with collagen-like domains. This binding triggers the ITIMs in LAIR1 to transmit inhibitory signals via SHP1 and SHP2 protein phosphatases that negatively regulate cellular activation (9–11). Notably, LAIR2 competes for collagen binding with LAIR1; LAIR2 therefore antagonizes LAIR1 inhibitory signaling, resulting in increased cellular activation (12). LAIR1 is expressed on immune cells, including cells of both the lymphoid and myeloid lineages, with the highest expression levels in monocytes and macrophages (13, 14).

Given their immunosuppressive function and high expression in some hematologic malignancies (15, 16), LAIR proteins have been evaluated as targets for cancer therapy. Any approach to targeting LAIR1-inhibitory signaling must address the differential effects of LAIR protein expression in cells to be restrained (leukemia/lymphoma, suppressive TAMs) and in cells to be activated (exhausted cytotoxic T cells), as well as the collagen content of the TME, because this also affects LAIR signaling. If the cancer cells express LAIR1, as in acute myeloid leukemia, agonism is more likely to be effective, as demonstrated by a LAIR1 agonist antibody that induced cell death in leukemia stem cells in cell and xenograft models (17). In contrast, loss or antagonism of LAIR1 signaling using LAIR1 KO, an anti-LAIR1 antagonist antibody, or a fusion of two LAIR2 molecules to an IgG1 backbone combined with checkpoint and, in some cases TGF-β blockade, has been shown to result in reduced tumor growth, increased infiltration of cytotoxic T cells, and remodeling of the extracellular matrix in models of solid tumors (breast, colon, lung) (18–21). Similarly, overexpression of LAIR2 or inhibition of SHP-1 activity sensitized resistant lung tumors to checkpoint blockade (22). These studies demonstrate that LAIR protein signaling is a promising target for cancer therapy.

## Combating LAIR1-mediated immunosuppression

**Figure 1:**
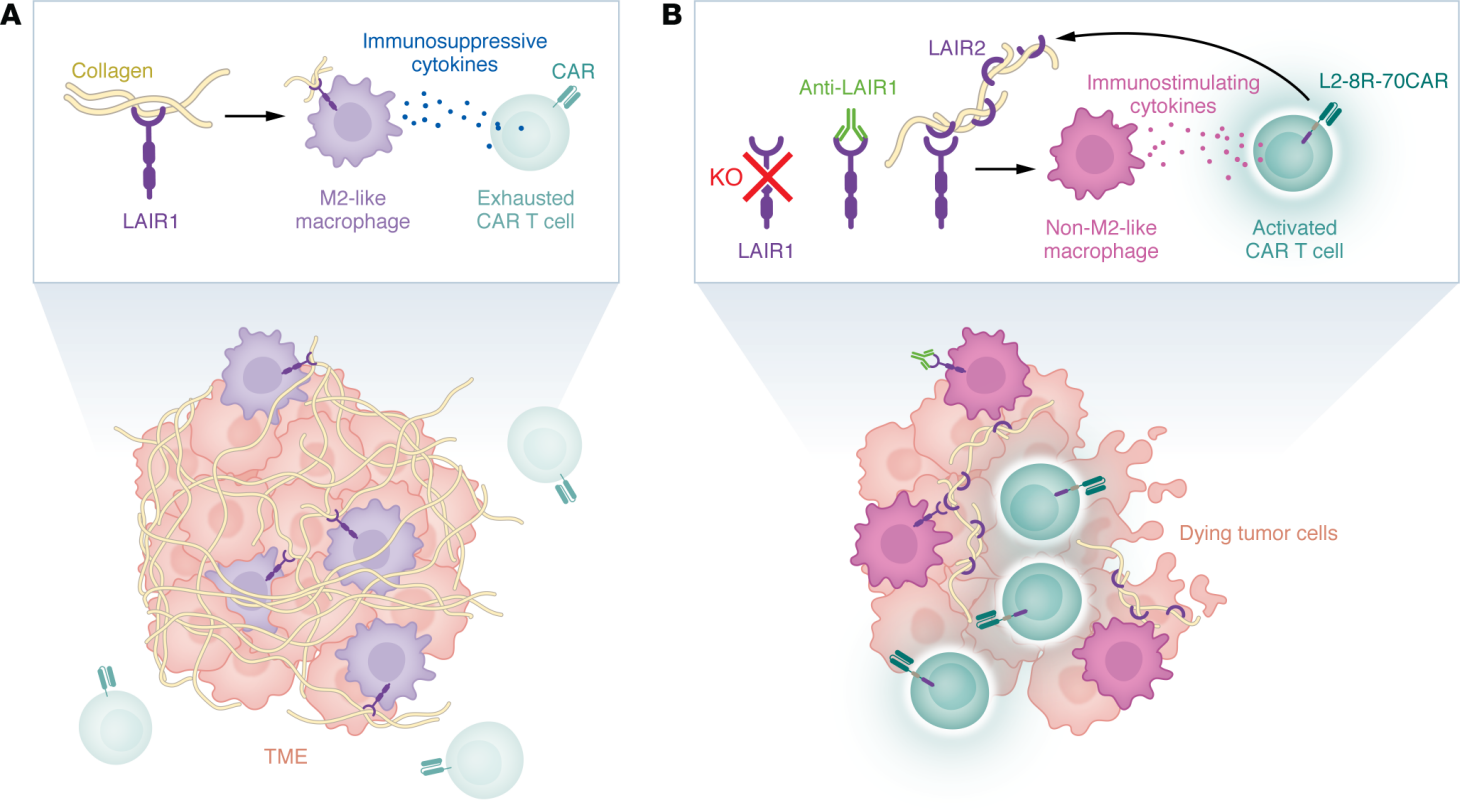
LAIR1-mediated suppression of the tumor immune response can be targeted by several strategies. (**A**) In tumors, LAIR1 expressed by macrophages binds collagen IV, promoting an M2-like phenotype and the production of immunosuppressive cytokines, which exhausts nearby T cells. Collagen IV production is increased within the TME, and CAR T cell penetration of the tumor is limited due to both exhaustion and the obstacle that the collagen network presents. (**B**) Treatments that block LAIR1 collagen binding (such as LAIR1 KO, antibodies against LAIR1, or LAIR1 antagonism via LAIR2 produced by the L2 module of the L2-8R-70CAR T cells) reduce M2 polarization and increase the production of immunostimulating cytokines, activating nearby T cells that attack and kill tumor cells, which are more accessible due to reduced collagen IV levels. These treatments increased survival in several different mouse tumor models.

In this issue of *JCI*, Tao and colleagues tested multiple approaches targeting LAIR1 signaling in several different in vitro human and in vivo mouse tumor models (4) (Figure 1). The authors’ interest in LAIR1 was inspired by the discovery of high LAIR1 expression in macrophages and microglia within human glioblastoma multiforme (GBM) tumors and M2-like TAMs in murine GBM models. They assessed the effect of LAIR1 loss on murine GBM models with and without coadministration of CD70 CAR T cells. LAIR1 KO alone increased the median overall survival (mOS) by 18%, decreased M2-like TAMs, and increased activated CD8^+^ T cells in the TME; the addition of CD70CAR T cells extended the mOS by nearly 50%, with similar findings in the TME. Turning to a human cell system with their IL-8 receptor/CD70CAR T (8R-70CAR T) cells and a CD70^+^ GBM cell line, the authors demonstrated that a commercially available anti–human LAIR1 antagonist antibody enhanced the CAR T antitumor response only in the presence of PBMC-derived M2-like macrophages. Treatment with a commercially available anti–mouse LAIR1 antibody alone or in combination with anti–PD-1 antibody or 8R70CAR T cells reduced tumor burden and extended mOS in murine GBM and lung carcinoma models. Outperformance of anti–PD-1 antibody and 8R-70 CAR T cells by anti-LAIR1 antibodies is remarkable and further reinforces the potential of anti-LAIR1 therapeutic approaches in patients with cancer.

Building on these results, the authors modified their 8R-70CAR construct to incorporate a module that enables secretion of LAIR2, which outcompetes LAIR1 for collagen binding, thus preempting its inhibitory signaling. In human T cells, this 3-in-1 L2-8R-70CAR substantially enhanced the antitumor response against human GBM cells in vitro. In a PD-1–resistant, immunocompetent mouse GBM tumor model, mouse T cells transduced with L2-8R-70CAR were superior to 8R-70CAR T cells, showing greater reductions in tumor size and extension of mOS. The striking synergistic effect warrants further study of LAIR2 secretion in additional CAR T models.

Because collagen is both the ligand of LAIR1 and a contributor to immunotherapy resistance, collagen content was evaluated in tumor models treated with anti-LAIR approaches. Mice with tumors treated with L2-8R-70CAR T cells or anti-LAIR1 antibody, as well as LAIR1-KO mice, exhibited lower levels of collagen IV staining within tumors — although there was no effect on collagen I levels. Notably, collagen levels in normal brain tissue were not affected by LAIR1 KO. Further analysis of anti-LAIR1–treated GBM revealed that there were fewer M2-like TAMs and lower expression of M2-like TAM signature genes, including *F13a1*, which encodes factor XIII-A (FXIII-A). Correlative expression of *LAIR1* and *F13A1* was observed in human GBM tumors, and expression of FXIII-A was reduced in *Lair1^–/–^* M2-like macrophages. The addition of FXIII-A during in vitro M2-like polarization of murine bone marrow monocytes resulted in increased levels of the M2-like marker Arg-1 in *Lair1^+/+^* but not *Lair1^–/–^* cells, which expressed minimal Arg-1 in either condition. Surprisingly, in vitro treatment of PD-1–resistant murine GBM cells with FXIII-A alone also led to increased collagen IV staining similar to that seen in untreated *Lair1^+/+^* GBM tumor–bearing mice. Together, these results support roles for FXIII-A and LAIR1 in promoting the formation of collagen IV networks in tumors, although further study is needed to define the mechanisms involved.

## Implications and future directions

The findings presented by Tao and colleagues address key outstanding questions in tumor immunosuppression and immunotherapy (4). First, their study underscores the important role of LAIR1 inhibitory signaling in promoting M2-like macrophage polarization, which has an overall suppressive effect on the antitumor immune response in M2-like TAMs, also demonstrated elsewhere (23). Second, their results reinforce other reports that blocking LAIR1 signaling reduces tumor growth, increases survival, and enhances antitumor immune responses. Other groups have also used LAIR1 KO, anti-LAIR1 antibodies, and LAIR2 to treat cancer (17–21). The innovative advancement from Tao and colleagues targets LAIR1 signaling by adding a LAIR2-secreting module to create a 3-in-1 CAR T cell that is substantially more effective than the previous CAR construct. Finally, the authors addressed a persistent question regarding LAIR protein function: How do LAIR1 signaling and collagen networks influence each other in the TME? They identified FXIII-A as an intriguing potential connection, although much remains to be elucidated.

Like all good science, the work by Tao and colleagues raises additional questions and new experimental directions. A logical first step is to add LAIR2 modules to other CAR constructs for testing in additional cancer types, particularly those characterized by immunosuppressive TAMs and dense collagen networks. The possible connection between FXIII-A and LAIR1 to modulation of collagen networks will require more mechanistic studies. While the authors showed that loss of LAIR1 resulted in decreased FXIII-A expression, loss of LAIR1 reduced M2-like polarization, thus the FXIII-A loss may simply reflect greater M1-like polarization. Another question involves the function of FXIII-A in the TME. FXIII-A is a promiscuous transglutaminase that can crosslink plasma fibrin, extracellular fibrin and fibronectin, and intracellular cytoskeletal proteins; however, a role for FXIII-A in collagen deposition has not been previously shown. Additional studies are needed to define the mechanism by which FXIII-A modulates collagen IV content in the TME, determine whether other collagen types are affected, and identify whether LAIR1 or FXIII-A can achieve this alone. Because FXIII-A is expressed by hematopoietic and mesenchymal cells, while LAIR1 is expressed by immune cells, tissue-specific Cre systems could be used to resolve this question. In summary, the compelling work from Tao et al. (4) extends our knowledge of LAIR1 signaling as an important regulator of the antitumor immune response and adds another weapon to our immunotherapy arsenal.

## Version 1 08/15/2025

Electronic publication
